# Pandemic Preparedness Against Influenza: DNA Vaccine for Rapid Relief

**DOI:** 10.3389/fimmu.2021.747032

**Published:** 2021-10-08

**Authors:** Tor Kristian Andersen, Johanna Bodin, Fredrik Oftung, Bjarne Bogen, Siri Mjaaland, Gunnveig Grødeland

**Affiliations:** ^1^ Department of Immunology and Transfusion Medicine, Institute of Clinical Medicine, University of Oslo, Oslo, Norway; ^2^ Division for Infection Control and Environmental Health, Norwegian Institute of Public Health, Oslo, Norway; ^3^ Department of Immunology and Transfusion Medicine, Clinic for Laboratory Medicine, Oslo University Hospital, Oslo, Norway

**Keywords:** pandemic, vaccine, DNA vaccine, APC, APC-targeting, influenza, inactivated vaccine

## Abstract

The 2009 “swine flu” pandemic outbreak demonstrated the limiting capacity for egg-based vaccines with respect to global vaccine supply within a timely fashion. New vaccine platforms that efficiently can quench pandemic influenza emergences are urgently needed. Since 2009, there has been a profound development of new vaccine platform technologies with respect to prophylactic use in the population, including DNA vaccines. These vaccines are particularly well suited for global pandemic responses as the DNA format is temperature stable and the production process is cheap and rapid. Here, we show that by targeting influenza antigens directly to antigen presenting cells (APC), DNA vaccine efficacy equals that of conventional technologies. A single dose of naked DNA encoding hemagglutinin (HA) from influenza/A/California/2009 (H1N1), linked to a targeting moiety directing the vaccine to major histocompatibility complex class II (MHCII) molecules, raised similar humoral immune responses as the adjuvanted split virion vaccine Pandemrix, widely administered in the 2009 pandemic. Both vaccine formats rapidly induced serum antibodies that could protect mice already 8 days after a single immunization, in contrast to the slower kinetics of a seasonal trivalent inactivated influenza vaccine (TIV). Importantly, the DNA vaccine also elicited cytotoxic T-cell responses that reduced morbidity after vaccination, in contrast to very limited T-cell responses seen after immunization with Pandemrix and TIV. These data demonstrate that DNA vaccines has the potential as a single dose platform vaccine, with rapid protective effects without the need for adjuvant, and confirms the relevance of naked DNA vaccines as candidates for pandemic preparedness.

## Introduction

Vaccines are highly efficient at prophylactic relief against infectious diseases, and vaccines against influenza, measles, and tuberculosis are examples of vaccines that annually save many lives (1, 2). The current SARS-CoV-2 pandemic has once again reminded us of the dependency on effective vaccines for control of a pandemic outbreak. In 2009, it became clear that the use of conventional influenza vaccines based on egg-production had several shortcomings. In particular, the production time was prolonged, hampering efficient use even in a situation where the correlate of protection was well established, and approved vaccines against influenza were easily available (3, 4). For pandemic control, rapid availability of well-matched vaccines is key (5–7).

In 2009, the conventional vaccines against pandemic influenza was produced within 6 months, which represents a record fast production for this vaccine format (8). Thus, it should be no surprise that the frontrunner vaccines developed against SARS-CoV-2 in 2020 were based on more versatile technologies (9–11). In this study, we have compared the immunogenicity and efficacy of conventional influenza vaccines to that of a novel DNA vaccine format. DNA vaccines are rapid to produce, easy to store and deploy independent of a cold chain, and highly versatile with respect to updating the vaccine to match new antigenic variants (12). Furthermore, the development of minimally invasive DNA vaccine delivery systems, such as microneedle patches (13, 14) or needle-free jet delivery (15, 16), makes naked DNA vaccines highly applicable in a mass vaccination scenario.

While DNA vaccines against influenza has been in development since the 1990’s with promising data in pre-clinical models, there are limited data from clinical trials due to low immunogenicity in larger animals (17). Some recent breakthrough has countered this, and most of the clinically approved DNA vaccines are based on delivery with viral vectors (11, 18, 19). While viral vector delivered DNA vaccines are attractive, the viral vector may in itself pose a risk for development of adverse events (20), and immune responses against the vector backbone may hamper repeated use, e.g. in prime boost vaccination schedules or updates for emerging viral variants.

Previously, we have developed a novel DNA vaccine format where the antigen was genetically linked to a targeting moiety specific for a selected receptor on antigen presenting cells (APC) (21–23). In brief, following delivery of naked DNA plasmids encoding the APC-targeted antigen under a single promotor, the cells at the injection site will secrete the corresponding proteins. The APC-specific targeting moiety will then direct the vaccine proteins specifically to the most relevant cells, and as such greatly enhance vaccine immunogenicity and efficacy (21–23). Of note, we have previously observed that steering of the APC-targeted vaccines to different APC receptors can polarize immune responses to either dominant antibody responses/Th2 or cellular responses/Th1 (24). As such, this vaccine platform could be tailored for enhanced induction of the most relevant correlate of protection for any disease (24–26). For influenza, the main correlate of protection against infection are neutralizing antibodies against the HA protein. We have previously found that targeting of HA to major histocompatibility complex class II (MHCII) molecules was superior at raising protective antibodies following vaccination, as compared to eight other APC specific targeting moieties (αCD11c, αCD40, Xcl-1, MIP-1α, FliC, GM-CSF, Flt-3L, αDEC205) (27). Hence, we have here used a plasmid encoding MHCII-targeted HA molecules for vaccination of mice (23, 28). Previously, such vaccination have demonstrated full protection against lethal influenza challenges in mice up to about a year after a single DNA vaccination (22, 24), as well as demonstrated promising efficacy in larger animals (23).

We have here compared the formation of immune responses in mice following vaccination with this MHCII-targeted DNA vaccine to that of conventional influenza vaccines. More specifically, we compared the MHCII-targeted DNA vaccine to Pandemrix, an adjuvanted inactivated split virion vaccine widely administered to counter the 2009 influenza pandemic, as well as the corresponding non-adjuvanted inactivated trivalent influenza vaccine (TIV) from the 2018/19 season.

We show that a single delivery of the MHCII-targeted DNA vaccine raised antibody responses similar to the adjuvanted Pandemrix, and both vaccines could offer long-lasting protection against a lethal influenza challenge. Interestingly, the MHCII-targeted DNA vaccine proved better than Pandemrix with respect to offering protection against a lethal influenza challenge one week after a single vaccination. This protection was likely attributed to the ability of the MHCII-targeted vaccine to also raise protective T cell responses.

## Materials and Methods

### Mice and Cell Lines

Female BALB/c mice aged 6-8 weeks (Janvier, le Genest-Saint-Isle, France) were used in all experiments. All experiments involving research animals were pre-approved for ethics by the Norwegian Food Safety Authority. Cell work was performed with human embryonic kidney 293E cells purchased from the American Type Culture Collection (ATCC; Manassas, VA, USA).

### Vaccines and Vaccination

Anesthetized mice [0.1mg/10g: cocktail of Zoletil Forte (250mg/ml; Virbac France), Rompun (20mg/ml; Bayer Animal Health GmbH), and fentanyl (50µg/ml; Actavis, Germany)] were vaccinated intra muscularly (i.m.) with 25µg DNA (αMHCII-HA) into each quadriceps femoris, immediately followed by electroporation over the injection site (Elgen; Inovio Biomedical Co., Blue Bell, PA). The αMHCII-HA plasmid encodes HA from influenza A/California/07/2009 (H1N1), aa 18-541, linked to the MHCII-specific scFv *via* a dimerization unit consisting of the C_H_3 domain of human IgG3 (22). All DNA vaccines were purified by using an EndoFree Plasmid Mega kit (catalog no. 12381; Qiagen, Hilden, Germany) and dissolved in sterile injection fluid (0.9% NaCl). Alternatively, anaesthetized mice were vaccinated i.m. with 1/10 human dose of Pandemrix with AS03 adjuvant (GlaxoSmithKline, Belgium), or a non-adjuvanted trivalent inactivated seasonal influenza vaccine [strains: A/Michigan/45/2015 (H1N1)pdm09-like virus, A/Singapore/INFIMH-16-0019/2016(H3N2)-like virus B/Colorado/06/2017-like virus (B/Victoria/2/87 lineage)].

### Viral Challenge

Mice were anaesthetized as described above, and a 5xLD_50_ dose of A/California/07/2009(H1N1) delivered in 10µl into each nostril. Mice were monitored daily for weight loss and euthanized at 80% of the original body weight. In figures, euthanized mice are scored as 80% for the remaining experimental time.

### Flow Cytometry and Imaging

Draining LNs (iliac) were harvested and single cell suspensions prepared by GentleMACS dissociator (Miltenyi Biotech, Germany). Cells were stained with anti-CD3 (75-0032, Tonbo biosciences, San Diego, CA, USA), anti-GL7 (144603, Tonbo), anti-CD38 (102718, Tonbo), and anti-B220 (552771, BD Biosciences, Franklin Lakes, NJ, USA). HA reactivity was evaluated by binding to a His-tagged recombinant HA (Cal07) protein with an Y98F substitution (29) (rec.HA^Y98F^), detected by anti-6xHis mAb (ab133714, Abcam, Cambridge, England). All samples were analyzed using an Attune NxT flow cytometer (Thermo Fisher Scientific, Waltham, MA, USA) and FlowJo software (ver.10).

Draining LNs were embedded in OCT mounting medium (00411243, Q Path, VWR, Radnor, PA, USA), immediately frozen on dry ice and stored at −80°C. Six-micrometer sections were collected on glass slides, air dried, fixed in room temperature acetone for 5 min, air dried, and blocked in 30% normal rat serum with FcRγ blocking reagent (10 μg/ml, HB-197). Sections were then incubated with 2µg/ml rec.HA^Y98F^, followed by rabbit anti-HA(Cal07) pAb (11085-T54, Sino Biological, Inc) and anti-GL7-PE (144608, BioLegend, San Diego, CA, USA). Finally, colors were amplified using anti-FITC-Alexa Fluor 488 (A-11090, Thermo Fisher Scientific) and anti-R Phycoerythrin-Texas Red (ab34734, Abcam), and counterstained with DAPI. Sections were mounted with ProLong Diamond Antifade Mountant (P36970, Thermo Fisher Scientific). Images were acquired in a Nikon Eclipse Ti microscope using a Nikon S Plan Fluor 20x objective with a 0.60 numerical aperture and a Nikon Digital Sight Camera. All micrographs were analyzed and processed using ImageJ Version: 2.0.0-rc-69/1.52p, Build: 269a0ad53f.

### Serum ELISA and Avidity Index ELISA

Blood was harvested by puncture of the saphenous vein, and sera collected by centrifugation. ELISA plates (Costar 5390, Corning, Corning, NY) were coated with 0.5µg/ml rec. HA from A/California/07/2009 (11085-V08H, Sino Biological, Inc., Wayne, PA, USA), blocked with 2% BSA/PBS, and incubated with serially diluted serum samples assayed for individual mice. Captured serum antibodies were detected with anti-mouse IgG1-bio (553500, BD Pharmingen, San Diego, CA, USA), or anti-mouse IgG2a- bio (553502, BD Pharmingen), and streptavidin-alkaline phosphatase (RPN1234, GE Healthcare, Buckinghamshire, UK), or alkaline phosphatase conjugated goat anti-mouse IgG (A2429, Saint-Louis, MO, USA). Plates were developed with phosphatase substrate (P4744, Sigma-Aldrich).

Resistance to UREA wash was used to calculate avidity index. Captured serum antibodies were incubated for 10min with 2M UREA or PBS before detecting remaining serum antibodies with alkaline phosphatase conjugated goat anti-mouse IgG (A9316, Sigma Aldrich). AUC was calculated for the dilution curves and baseline for AUC was calculated based on NaCl serum levels. Avidity index is defined as AUC for samples treated with UREA divided by AUC for the corresponding PBS treated sample.

### ELISpot Assay

Bone marrow was harvested from femur and tibia. Single cell suspensions were prepared and seeded on MultiScreen HTS filter plates (MSIPS45, Merck Millipore Ltd., Tullagreen, Ireland) pre-coated overnight at 4°C with 0.5 μg/well of rec.HA (Cal07) (11085-V08H, Sino Biological), and incubated for 20h. Spots were detected with anti-mouse IgG (A1418, Sigma-Aldrich), developed with phosphatase substrate (P4744, Sigma-Aldrich) and analyzed in CTL- ImmunoSpot^®^ analyzer (CTL, Shaker Heights, OH, USA).

### *In Vivo* Cellular Cytotoxicity Assay

*In vivo* cellular cytotoxicity assay were adapted from Durward et al. (30). In brief, splenocytes were harvested and single cell suspensions prepared. Splenocytes were incubated with the MHC class I restricted influenza HA (Cal07) peptide IYSTVASSL, the NP peptide (RLIQNSLTIERMVLS), or a negative control peptide, at a density of 5x10^7^ cells/mL for 1 h at 4°C followed by 30 min incubation at 37°C. Peptide‐loaded cells were washed twice in PBS and subsequently stained with 5 μM CellTrace Violet (CTV) (C34557, Life Technologies) (HA peptide loaded cells), or 1 μM CellTrace Far Red (CTFR) (C34564, Life Technologies) (NP peptide loaded), or double stain (CTV and CTFR) (negative control) at a density of 5x10^7^ cells/mL for 20 min at 37°C. Cells were mixed in equal ratios (1:1:1), and a total of 15x10^6^ cells injected i.v. in a 100µl volume to vaccinated mice. Spleens were harvested 16h later, single cell suspensions prepared, and the presence of peptide loaded cells investigated by flow cytometry. The ratio of CTV to CTV/CTFR or CTFR to CTV/CTFR cells were calculated as % specific lysis = [1 − (average ratio in group with NaCl vaccinated mice/experimental ratio)].

### *In Vitro* T Cell Stimulation and Cytokine Staining

Spleens from mice were collected 9 and 21 days after vaccination and homogenized through a wire mesh to get a single cell suspension by Lympholyte M (Cedarlane, Burlington, US) gradient centrifugation, and thereafter kept frozen in Fetal Bovine Serum (FBS, Sigma/Merck) with 10% DMSO (Sigma/Merck) at -150°C. Splenocytes were thawed, washed in RPMI 1640 (Gibco, Thermo Fischer Scientific) with 10% FBS (Sigma/Merck) and rested for 24 hours prior to stimulation with 5.6 µg HA/ml (400HAU) of A/California/07/2009 (H1N1) for 4 hours at 37°C in the presence of 2.5 µg/ml brefeldin A (BFA, Sigma/Merck). Positive control was stimulated with 50 ng/ml phorbol myristate acetate (PMA, Sigma/Merck) and 1 µg/ml ionomycin (Sigma/Merck). Cells were stained for viability (Live/dead aqua, Molecular Probes, Thermo Fischer Scientific) and extracellular markers in Brilliant staining buffer (BD Biosciences, San Antonia, CA, US) (each staining for 30 minutes at room temperature), then fixed and permeabilized (45 minutes at 4°C using Foxp3 fixation and permeabilization kit, eBioscience) prior to intracellular staining (1 hour 4°C). Percentage of positive CD3, CD4, CD8, CD44, CD62L, CD25, CD19, CD49b, Foxp3, CD107a, IFNγ, TNFα, IL-2 and IL-17A cells was analysed on a ZE5 flow cytometer (Bio-Rad, CA, US). Fractions of memory T cells; T effector memory (TEM) CD44^+^CD62L^-^, and T central memory (TCM) CD44^+^CD62L^+^, and naïve T cells CD44^-^CD62L^+^ as well as NK and NKT cells were also assessed using FlowJo_V10 (Tree Star, San Carlos, CA, US).

Splenocytes were harvested 21 days after vaccination, and single cell suspensions rested for 24 hours prior to stimulation with 5.6 µg HA/ml (400HAU) A/California/07/2009 (H1N1) for 4 hours at 37°C in the presence of 2.5 µg/ml brefeldin A (BFA, Sigma). Splenocytes were then stained for identification of IFNγ, IL-2, and TNFα positive CD4 and CD8 T-cells.

### Statistical Analysis

The p-values represent exact values calculated by unpaired non-parametric two-tailed Mann-Whitney tests. Weight curves were analyzed with two-way ANOVA, and survival curves with the Gehan-Breslow-Wilcoxon test. Statistical analysis of flow cytometry was performed using one-way ANOVA with the Holm-Sidiak multiple-comparison test. All analysis was performed using GraphPad Prim 9 software.

## Results

### Induction of Strong and Long Lasting Protective Antibodies up to 6 Months After a Single Vaccination

In order to compare the antibody kinetics of different vaccine strategies, we vaccinated mice once i.m. with either TIV, Pandemrix, or the MHCII-targeted DNA vaccine (αMHCII-HA). Both Pandemrix and αMHCII-HA are monovalent vaccines, designed to protect against A/California/07/2009 (H1N1), whereas TIV in addition to a pdm09 like strain contains an H3N2 strain and an influenza B strain. The plasmids encoding αMHCII-HA were formulated in a physiological saline solution (NaCl), while Pandemrix was formulated with the adjuvant solution AS03. Thus, both saline and AS03 were used as experimental negative controls.

**Figure 1:**
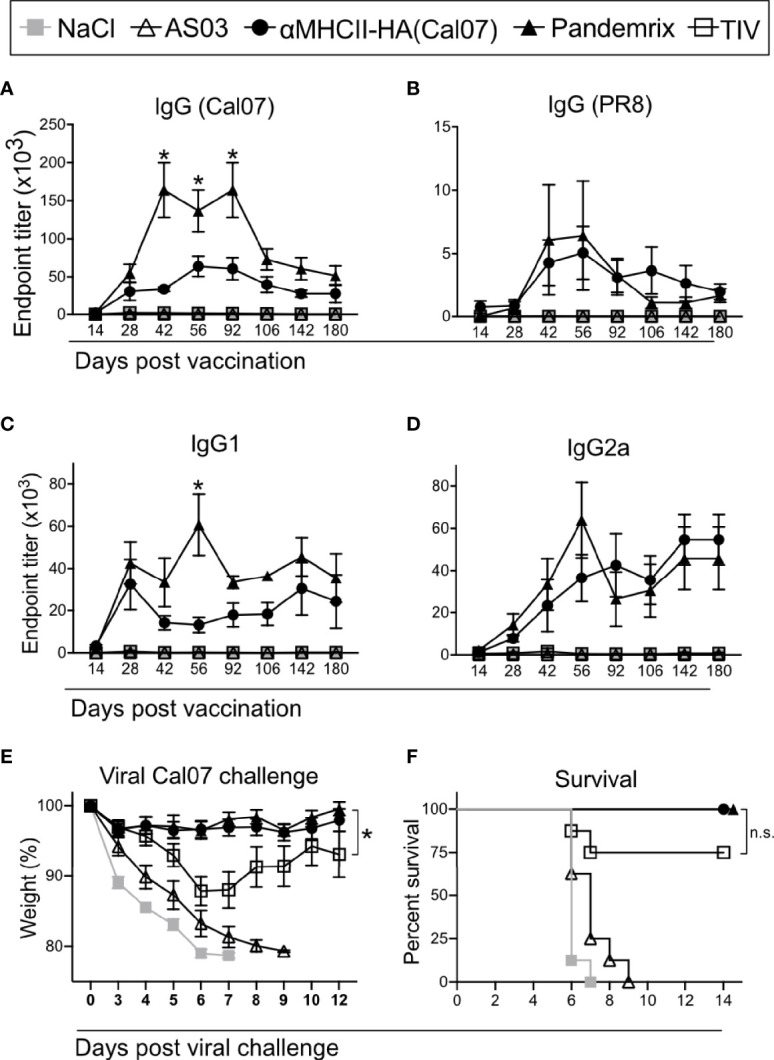
Long term antibody responses and protection after a single vaccination. Mice (n=8/group) were vaccinated i.m. with the indicated vaccines. **(A–D)** Serum antibodies were monitored up to 6 months after vaccination in ELISA for **(A)** total IgG against HA from Cal07, **(B)** total IgG against HA from PR8, **(C)** IgG1 against HA from Cal07, and **(D)** IgG2a against HA from Cal07. *P < 0.05 for αMHCII-HA versus Pandemrix. **(E, F)** At 180 days after vaccination, mice were challenged with 5xLD_50_ dose of Cal07. **(E)** Weight was monitored. **(F)** Survival curve after challenge, defined by 20% weight loss. **(A–E)** Data shown are mean ± SEM, *P < 0.05 (two-way ANOVA) **(F)** *P < 0.05 (Gehan-Breslow-Wilcoxon test). n.s., not significant.

Following a single vaccination with the different vaccines, serum samples were collected and monitored for HA specific antibodies over 180 days. Both αMHCII-HA and Pandemrix rapidly generated high titers of Cal07 HA specific IgG that were maintained over time. A peak was observed between 42 and 92 days post vaccination, and where the mice vaccinated with Pandemrix had significantly higher total IgG levels as compared to αMHCII-HA (**Figure 1A**). However, αMHCII-HA raised significantly higher antibody responses as compared to TIV, which only induced modest antibody responses in this system. After the peak, the responses seemed to reach a plateau from about day 106, and that were maintained for at least 180 days.

The antibody responses were highly strain specific, and only mild reactivity against the serologically different strain A/Puerto Rico/8/1934 (H1N1) (PR8) was detected after vaccination with Pandemrix or αMHCII-HA (**Figure 1B**). Interestingly, the difference between Pandemrix and αMHCII-HA seemed to be mostly due to a significantly higher amount of IgG1 antibodies following vaccination with Pandemrix (**Figure 1C**), while there were no significant differences in IgG2a levels (**Figure 1D**).

At day 180, mice were challenged with a lethal dose of influenza virus Cal07. Weight was monitored and used as an objective indicator of morbidity. As expected from the measured antibody responses, both αMHCII-HA and Pandemrix vaccination induced significantly improved protection as compared to TIV, characterized by near sterile protection and minimal weight loss after challenge (**Figure 1E**). In accordance with ethical requirements, mice that reached a 20% weight loss during the infection were euthanized. Importantly, none of the mice vaccinated with αMHCII-HA or Pandemrix reached this threshold (**Figure 1F**). Based on the low antibody levels associated with TIV, it may, however, be surprising that only 2/8 mice in this group lost 20% or more of their weight, as opposed to the negative control groups where 8/8 had to be euthanized. The mice receiving TIV lost weight until day 6 after infection, but from then on stabilized and regained weight (**Figure 1F**).

In sum, we observed that a single vaccination with either αMHCII-HA or Pandemrix could raise strong and long-lasting strain specific protective antibody responses against HA.

### Rapid Induction of Antibodies and Protection Against a Lethal Challenge With Influenza Virus

**Figure 2:**
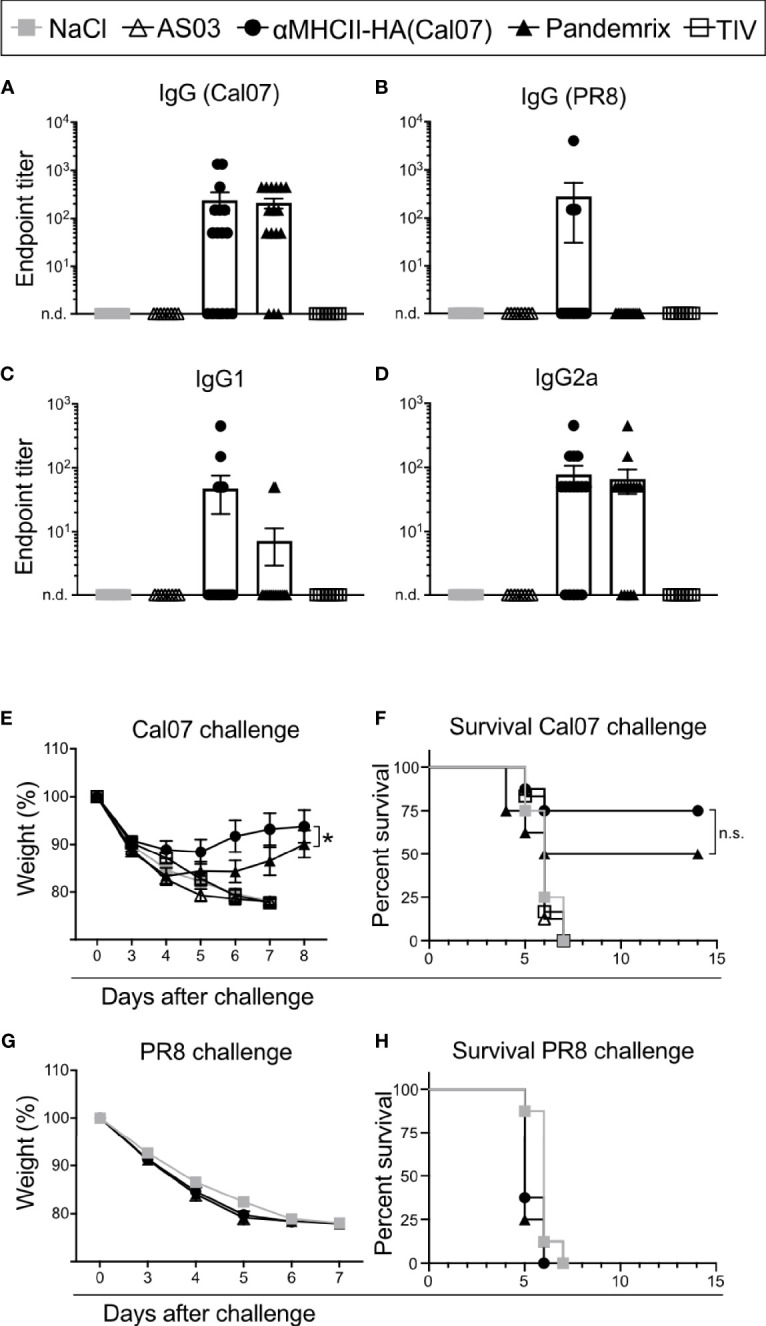
Rapid induction of antibodies and protection after vaccination. Mice were vaccinated i.m. with the indicated vaccines. **(A–D)** Serum antibodies were monitored at day 7 after vaccination in ELISA for **(A)** total IgG against HA from Cal07, **(B)** total IgG against HA from PR8, **(C)** IgG1 against HA from Cal07, and **(D)** IgG2a against HA from Cal07. Pandemrix, αMHCII-HA, and NaCl: n=16/group. AS03 and TIV: n=8/group. **(E, F)** At day 8 after vaccination, mice (n=8/group) were challenged with a 5xLD_50_ dose of influenza Cal07. **(E)** Weight was monitored. **(F)** Survival curve, as defined by a 20% weight loss. **(G, H)** At day 8 after vaccination, mice (n=8/group) were challenged with a 5xLD_50_ dose of influenza PR8. **(G)** Weight was monitored. **(F)** Survival curve, as defined by a 20% weight loss. **(A, D, E, G)** Data shown are mean ± SEM, *P < 0.05 (two-way ANOVA); **(F, H)** *P < 0.05 (Gehan-Breslow-Wilcoxon test). n.s., not significant.

Time is essential during a pandemic outbreak, with respect to both production time and the generation of protective immunity. Thus, we investigated how fast the different vaccines were able to induce protective immunity. BALB/c mice were vaccinated once with αMHCII-HA, Pandemrix, TIV, or controls, and sera examined for antibody responses at day 7 after vaccination. Importantly, a majority of the mice vaccinated with either αMHCII-HA or Pandemrix had detectable levels of HA specific IgG, but there were also some that had not yet seroconverted (**Figure 2A**). TIV immunized mice did not display any serum antibodies at day 7. Interestingly, αMHCII-HA was the only vaccine that could induced any antibody responses against the heterologous strain PR8, albeit only in 2 out of 16 mice in the group (**Figure 2B**). For homologous antibody responses against HA from Cal07, IgG1 and IgG2a levels were similar for Pandemrix and αMHCII-HA, but as was observed for total IgG, not all mice had seroconverted at this early time point (**Figures 2C, D**).

At day 8 after a single vaccination, mice were given a lethal dose of influenza Cal07 virus and weight was monitored. Interestingly, mice receiving αMHCII-HA lost significantly less weight as compared to mice vaccinated with Pandemrix, but there was a smaller initial weight loss also in this group (**Figure 2E**). The trend also held when assessing survival as defined by a 20% weight loss, with αMHCII-HA displaying a 25% relative improved survival rate, albeit not statistically significant, when compared to Pandemrix (**Figure 2F**). Mice vaccinated with TIV were not protected 7 days after challenge. None of the vaccines induced protection against the heterologous PR8 virus at day 8 post vaccination (**Figures 2G, H**).

Taken together, we found that a single vaccination with either Pandemrix or αMHCII-HA could rapidly lead to seroconversion that translated into protection already one week after vaccination. In addition, vaccination with the MHCII-targeted DNA vaccine significantly improved morbidity as compared to Pandemrix.

### Plasma Cells and High Avidity Antibodies After Vaccination

**Figure 3:**
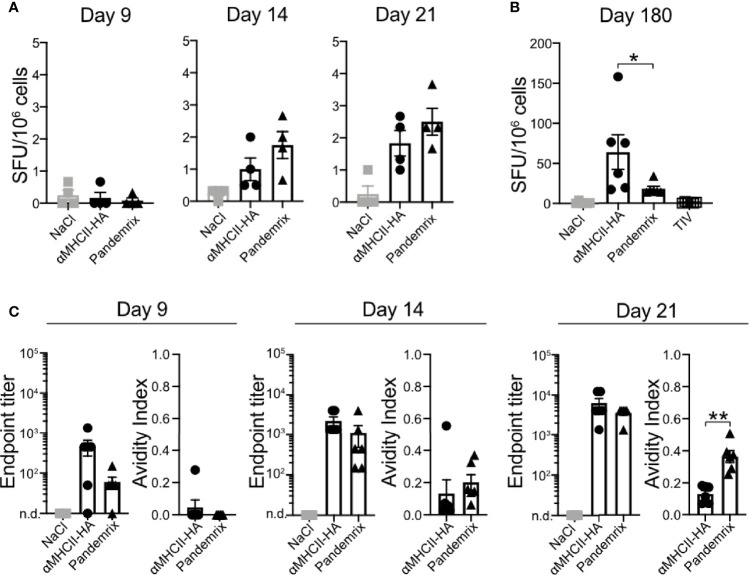
Plasma cells in bone marrow and antibody avidity after vaccination. Mice (n=6/group) were vaccinated i.m. with the indicated vaccines. **(A, B)** Bone marrow (tibia) was harvested, and anti-HA secreting cells examined by ELISpot at **(A)** days 9, 14, and 21 after vaccination, or **(B)** day 180 post vaccination. **(C)** Antibody titers and avidity index of serum antibodies against HA as measured in ELISA from the indicated time points post vaccination. Data shown are mean ± SEM, *P < 0.05, **P < 0.01 (Mann-Whitney).

To characterize the antibody response induced after vaccination with the different vaccines in detail, we first investigated the presence of plasma cells in bone marrow following vaccination. Thus, bone marrow was harvested from mice that had been vaccinated once with αMHCII-HA or Pandemrix. Single cell suspensions were prepared, and the number of anti-HA (Cal07) secreting cells assayed by ELISpot. At day 9, we detected no anti-HA secreting cells in bone marrow, but by day 14 anti-HA secreting cells had formed for both these vaccines. Although low, at day 21 the levels had doubled, indicating a steady rise in anti-HA secreting cells in response to vaccination. The development was similar for both αMHCII-HA and Pandemrix, but there was a tendency that Pandemrix had slightly higher numbers of plasma cells at day 14 and 21 (**Figure 3A**).

To evaluate long-term responses, we also examined plasma cells at day 180 in bone marrow following vaccination with TIV, αMHCII-HA, and Pandemrix. Interestingly, mice vaccinated with αMHCII-HA had significantly higher levels of plasma cells in the bone marrow as compared to mice receiving Pandemrix. Mice vaccinated with TIV did not show any plasma cells in response to vaccination after 180 days (**Figure 3B**).

Next, we wanted to investigate the avidity of the vaccine induced antibodies and set up an assay measuring the resistance to UREA wash as an indication of antibody binding avidity. In this assay, relative signals between washing with UREA or PBS in ELISA were compared, and an avidity index of 0 or 1 indicated no resistance or absolute resistance to UREA wash, respectively. Sera collected from mice at day 7, 14, and 21 post vaccination were assayed. In accordance with the above results from ELISA (**Figures 1**, **2**), we did not observe significant differences in antibody titers for Pandemrix and αMHCII-HA. However, mice vaccinated with Pandemrix demonstrated a steady increase in serum antibodies with higher avidity towards HA from Cal07 (**Figure 3C**), mimicking the tendency observed for plasma cells in bone marrow (**Figure 3A**). At day 21, mice vaccinated with Pandemrix had significantly more serum antibodies with increased avidity as compared to mice vaccinated with αMHCII-HA, even though serum antibody levels were similar between the two vaccines (**Figure 3C**).

In sum, we found that Pandemrix induced antibodies with higher avidity more rapidly than αMHCII-HA (day 21). However, with time (6 months) the level of plasma cells in response to αMHCII-HA vaccination was significantly higher than after vaccination with Pandemrix.

### Germinal Center Induction After Vaccination and HA Reactive B Cells

The early presence of plasma cells in bone marrow could indicate a strong germinal center (GC) reaction to the vaccine antigen (**Figure 3A**). Thus, we wanted to investigate the formation of GCs, as well as the presence of HA reactive B cells with a GC phenotype.

**Figure 4:**
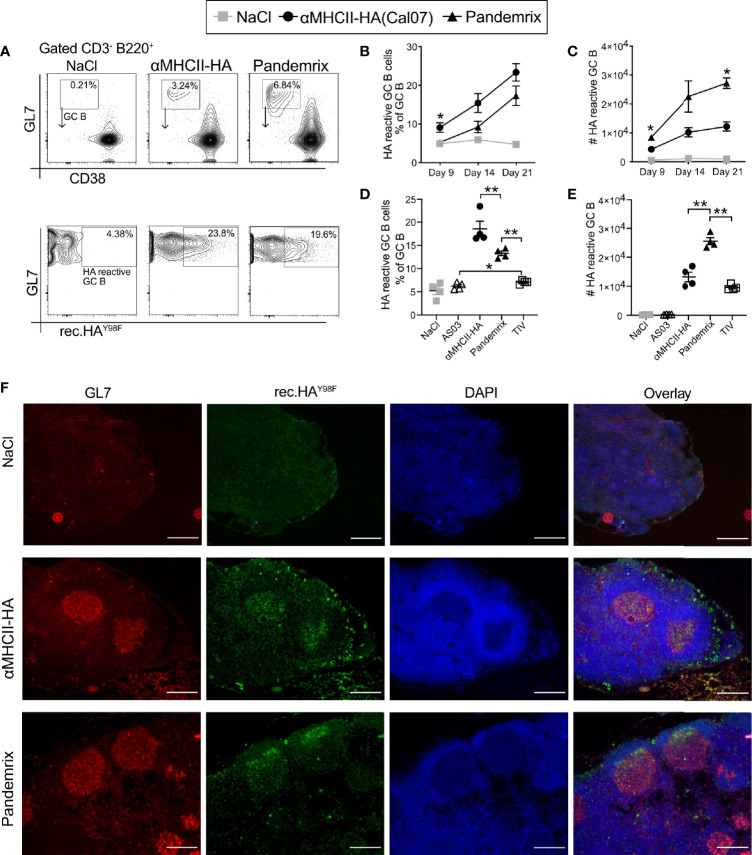
Germinal center response and early formation of plasma cells after vaccination. Mice (n=4/group) were vaccinated i.m. with the indicated vaccines. **(A–E)** Draining lymph nodes (iliac) were harvested and examined for B cells (CD3^-^ B220^+^), GC markers (GL7^+^CD38^lo^), and binding to a recombinant HA probe by flow cytometry. **(A)** Gating strategy. Representative flow charts are shown. **(B)** Fraction of HA reactive B cells with a GC phenotype on the indicated time points. **(C)** Absolute numbers of HA reactive GC B cells from draining LNs at the indicated time points. **(D, E)** In a new experiment, the GC response was investigated at 21 days post vaccination. **(D)** Fraction of HA reactive B cells with a GC phenotype. **(E)** Absolute numbers of HA reactive GC B cells. **(F)** Cryosections of draining lymph nodes harvested 21 days after vaccinations, and stained with GL7 (red), rHA^Y98F^ (green), and DAPI (blue). Scale bar is 200µm. Data shown are mean ± SEM, *P < 0.05, **P < 0.01 (Mann-Whitney).

Mice were vaccinated once and draining lymph nodes (LN) (iliac) harvested on days 9, 14, and 21. Cells from the prepared single cell suspensions were stained for GC B cells (B220^+^ CD38^lo^ GL7^+^), identified by a recombinant HA probe (**Figure 4A**). A steady rise in HA reactivity among B cells was observed from day 9 through day 21 post vaccination (**Figure 4B**). Mice receiving αMHCII-HA had GC B cells with a significantly elevated HA reactivity. However, Pandemrix induced a stronger GC reaction, and although the percentage of GC HA reactivity was lower in these mice, the total number of HA reactive GC B cells was significantly higher than after vaccination with αMHCII-HA (**Figure 4C**).

The increased levels of GC B cells observed after vaccination with Pandemrix was likely augmented by the adjuvant AS03. Pandemrix is a split vaccine and also contains other antigens than HA. Thus, a new experiment was performed for day 21 post vaccination to also include an adjuvant control group and the non-adjuvanted split vaccine TIV. As expected, vaccination with αMHCII-HA again induced the highest percentages of HA reactive B cells with a GC phenotype in the LNs. The control groups vaccinated with NaCl or AS03 defined the background, and we observed that TIV induced low, but significant, levels of HA reactive GC B cells (**Figure 4D**). When looking at the total number of HA reactive GC B cells, however, we again observed that Pandemrix induced the highest absolute numbers (**Figure 4E**).

The formation of GC was also evaluated by microscopic imaging of LNs harvested at day 21 after vaccination. The cryopreserved LN sections were stained with the GC activation marker GL7, the recombinant HA probe, and DAPI (**Figure 4F**). Multiple GC structures with HA reactivity were observed for the mice receiving αMHCII-HA or Pandemrix, in accordance with the flow cytometry data.

Taken together, the data demonstrate that vaccination with αMHCII-HA induced a response where the reactivity of GC B cells was focused on HA. Pandemrix induce a stronger immune response in total, and as such had a higher total number of GC B cells and HA reactive GC B cells.

### Strong Cytotoxic T-Cell Responses Induced by αMHCII-HA

**Figure 5:**
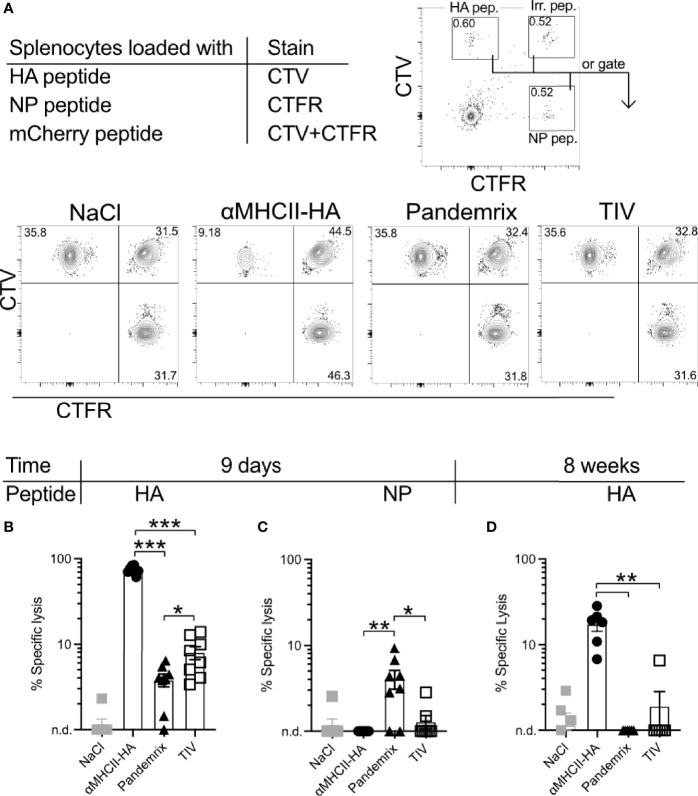
Short and long-term antigen-specific cytotoxic responses after vaccination. Mice (n=6-8/group) were vaccinated once i.m. with the indicated vaccines. After 9 days or 8 weeks, splenocytes from naïve mice were loaded with MHC class I restricted peptides from HA (IYSTVASSL), NP (RLIQNSLTIERMVLS), or irrelevant peptide. Cells were stained with CellTrace Violet (CTV), CellTrace Far Red (CTFR), or double stained with CTV and CTFR, respectively. Cells were mixed in equal ratios and injected i.v. to vaccinated mice. 16h later, spleens were harvested and the presence of peptide loaded splenocytes were assessed by flow cytometry. **(A)** Gating strategy for identification of transferred cells. **(B)** HA-specific lysis of splenocytes 9 days after vaccination. **(C)** NP-specific lysis of splenocytes 9 days after vaccination. **(D)** HA-specific lysis of splenocytes 8 weeks after vaccination. Data shown are mean ± SEM, *P < 0.05, **P < 0.01, ***P < 0.001 (Mann-Whitney).

The fairly similar B cell activation and antibody levels observed after vaccination with Pandemrix or αMHCII-HA led to the question of whether differences in induction of cytotoxic T cells could explain the improved survival that was observed in mice at day 8 after vaccination with αMHCII-HA (**Figure 2**). Thus, single cell suspensions of splenocytes from naïve mice were loaded with MHC class I restricted peptides from HA, influenza nucleoprotein (NP), or an irrelevant peptide as negative control, and stained with cell trace dyes. Next, the peptide loaded splenocytes were injected i.p. into mice immunized 9 days or 8 weeks prior with TIV, Pandemrix, αMHCII-HA, or NaCl. Following a 16h incubation, spleens were harvested and the presence of transferred cells investigated by flow cytometry (**Figure 5A**). The ratios of HA or NP peptide loaded splenocytes to the irrelevant peptide negative control in NaCl treated mice was used as a reference to calculate the relative specific lysis of HA or NP peptide loaded cells in the vaccinated groups.

Importantly, mice vaccinated 9 days earlier with αMHCII-HA displayed a strong cytotoxic response towards the HA peptide that was about 10-fold higher than that observed in mice vaccinated with Pandemrix (**Figure 5B**). Vaccination with Pandemrix raised a similar cytotoxic response against both NP and HA, while αMHCII-HA, as expected, did not induce any cytotoxic activity towards the NP peptide (**Figure 5C**). Interestingly, TIV induced similar responses as Pandemrix against both groups of peptides. In order to also examine the long-term cytotoxic potential, we set up a similar experiment 8 weeks post vaccination. The cytotoxic responses were markedly reduced in all the vaccine groups as compared to day 9, but the group vaccinated with αMHCII-HA maintained a significant and strong cytotoxic response (**Figure 5D**).

In sum, the data clearly demonstrated that αMHCII-HA induced a superior cytotoxic response both at early and later time points as compared to Pandemrix. The early induction of cytotoxic immunity could explain the improved protection observed after vaccination with αMHCII-HA and the viral challenge at 8 days post vaccination (**Figure 2**).

### Cytokine Secretion Following Vaccination

**Figure 6:**
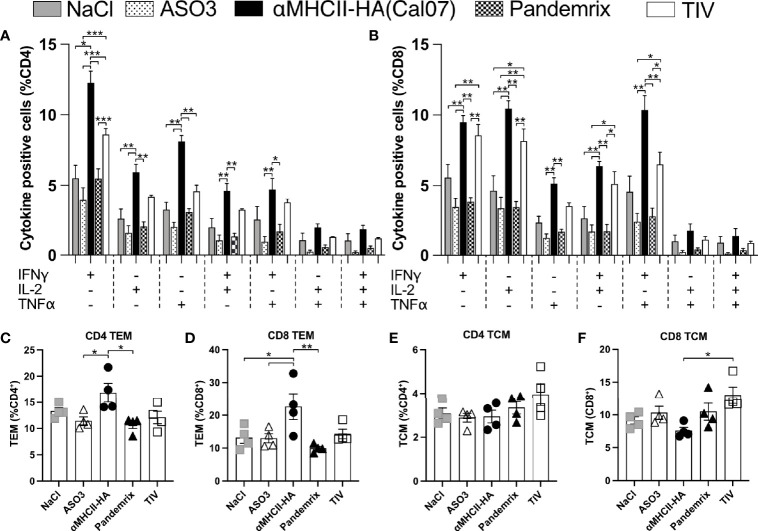
Multifunctional T cell subsets after vaccination. Mice (n=4/group) were vaccinated once i.m. with the indicated vaccines. 21 days after vaccination, splenocytes were harvested, and following overnight resting stimulated for 4h with HA (Cal07) in the presence of a protein transport inhibitor. Single, double, or triple expression of cytokines IFNγ, IL-2, and TNFα were investigated for CD4 **(A)** and CD8 **(B)** T cell subsets. **(C, D)** Effector memory (T_EM_) and central memory (T_CM_) T cell subsets were defined as CD44^+^ CD64L^-^, and CD44^+^ CD64L^+^, respectively. **(C)** Presence of CD4 T_EM_. **(D)** Presence of CD8 T_EM_. **(E)** Presence of CD4 T_CM_. **(F)** Presence of CD8 T_CM_. Data shown are mean ± SEM, *P < 0.05, **P < 0.01, ***P < 0.001 (Anova og Tukey’s multiple comparison).

T cells with an enhanced effector function have often been characterized by the dual secretion of two or more key cytokines (31), and the significant difference observed for cytotoxic responses between the vaccine groups (**Figure 5**) points towards different functional T cell profiles. Thus, we investigated the secretion of IFNγ, IL-2, and TNFα in T cells from splenocytes harvested 21 days after a single vaccination, and stimulated *ex vivo* with HA (Cal07). In accordance with the improved cytotoxic response following vaccination with αMHCII-HA (**Figure 5**), the highest numbers of IFNγ, IL-2, and TNFα secreting CD4 T-cells were observed for this group (**Figure 6A**). TIV induced somewhat higher numbers of cytokine secreting cells as compared to Pandemrix. The trend held also for double secreting CD4 T-cells. Triple secreting CD4 T-cells were not observed for any vaccine groups (**Figure 6A**). A similar trend was observed for the CD8 T-cells, but with somewhat higher levels (**Figure 6B**).

While the cytokine profiles indicated an increased level of effector T-cells, analysis of cells with effector memory (T_EM_) and central memory (T_CM_) phenotype was performed for CD4 and CD8 T-cells after *ex vivo* antigen stimulation. T_EM_ was identified as CD4/CD8^+^ CD44^+^ CD64L^-^ cells and T_CM_ was identified as CD4/CD8^+^ CD44^+^ CD64L^+^ cells. The T_EM_ subsets were slightly elevated in mice receiving αMHCII-HA for both CD4 and CD8 T-cells, whereas the other vaccine groups were similar to background (**Figures 6C, D**). T_CM_ were not clearly elevated above background levels (NaCl and AS03), although the percentage of CD8 T_CM_ seemed to be higher in mice receiving TIV compared to αMHCII-HA (**Figures 6E, F**).

Taken together, the data confirms an increased potential for activation of multifunctional T cells following vaccination with αMHCII-HA, as compared to Pandemrix and TIV. Further, immunization with αMHCII-HA increased the T_EM_ levels of both CD4 and CD8 T cells, indicating a strong protective effect. This is reflected in the highest survival rate after viral challenge compared across the vaccine platforms tested.

## Discussion

The current SARS-CoV-2 pandemic has once again reminded us of our dependency on effective vaccines for control of a pandemic outbreak, and new vaccine platforms that efficiently can quench pandemic emergences are urgently needed. It is particularly important that the vaccines can be rapidly available for deployment in the population, and that protective immune responses are raised rapidly after vaccination.

In this study, we compared formation of immune responses in mice following vaccination with an MHCII-targeted DNA vaccine to that of conventional influenza vaccines. More specifically, we used an adjuvanted, inactivated split virion vaccine widely administered to counter the 2009 influenza pandemic (Pandemrix) and the corresponding conventional non-adjuvanted seasonal trivalent inactivated vaccine (TIV). A single delivery of the MHCII-targeted DNA vaccine raised HA-specific antibody responses with high avidity already after one week, similar to the responses induced by the adjuvanted Pandemrix, and both vaccines offered long-lasting protection against a lethal influenza challenge. Interestingly, the MHCII-targeted DNA vaccine proved even better than Pandemrix with respect to offering early protection against a lethal influenza challenge, probably due to the enhanced activation of cellular immunity after vaccination with αMHCII-HA.

DNA vaccines against influenza have been in development since the 1990’s with promising data in pre-clinical models, but the reduced efficacy often observed in larger animals and humans has hampered progression to clinical application (17). New formulations such as lipid nanoparticles (32, 33), viral vector formulations (34, 35), and gene delivery methods (36) have increased vaccine efficacy, and a naked DNA vaccine from Inovio against SARS-CoV-2 just entered Phase 3 clinical testing (37).

The DNA vaccine format has several advantages for use in a pandemic setting, including advantageous price points and a cold chain independent distribution. A vaccine suited for pandemic preparedness against influenza should also be easily adaptable to match antigens of emerging strains and perform consistent across known influenza subtypes. Importantly, we have previously developed MHCII targeted DNA vaccines against several influenza subtypes, and observed a consistent and high immunogenicity in mice and larger animals (22, 23, 38, 39).

Besides the advantages of the DNA vaccine format, large-scale use of prophylactic DNA vaccines have also raised some safety concerns. Most pronounced is perhaps the potential for integration into host genomes (40, 41), antibiotic resistance (42), and DNA directed auto immunity (43). Fortunately, these phenomena have not been detected during clinical trials (37, 44–47). DNA vaccines have thus far demonstrated a good safety profile during clinical evaluations, but, as we have recently been reminded (48), rare adverse events are difficult to detect prior to use in a larger population.

The main correlate of protection against influenza is neutralizing antibodies that can block viral entry, and influenza vaccines typically aim for induction of these. Here, the rapid rise observed in antibody levels after vaccination with αMHCII-HA or Pandemrix was supported by increases in GC B-cells and plasma cells in bone marrow. These parameters were also reflected by the avidity index scores of serum antibodies for the two vaccines. Pandemrix induced a slightly stronger response than the DNA vaccine for practically all antibody or B-cell related measurements at early time points, although not statistically significant. Further, Pandemrix induced a very high number of GC B-cells, but the total HA reactivity was increased with αMHCII-HA. The adjuvant AS03 likely contributed to the high number of GC B cells found in draining lymph nodes after Pandemrix vaccination. AS03 is optimized for increased influx of immune cells to lymph nodes and B cell recruitment (49), and presumably contributed to a significant increase in the number of HA reactive GC B cells and the tendency of higher plasma cell numbers in the bone marrow at similar time points. Fluorescent micrographs demonstrated that the GCs with HA reactivity were slightly enhanced following vaccination with Pandemrix as compared to αMHCII-HA, in general accordance with GC B cell profile seen in the flow cytometry data (**Figure 4**).

Importantly, both Pandemrix and αMHCII-HA induced full protection against a lethal influenza challenge at 180 days post a single vaccination, with virtually no weight loss observed. Thus, the elevated IgG1 responses observed after vaccination with Pandemrix (**Figure 1**), as well as the increased avidity observed during the first weeks after vaccination (**Figure 3**), did not make a difference for the protective capacity long- or short-term as compared to MHCII-HA (**Figures 1**, **2**). At day 180 post a single vaccination, we also observed lower levels of anti-HA secreting plasma cells in bone marrow after Pandemrix vaccination as compared to αMHCII-HA. This also did not hamper protection, indicating that both vaccines were able to induce sufficient memory formation. TIV induced moderate long-term protection, in accordance with expectations for the single delivery of a non-adjuvanted vaccine administered to naïve mice.

In a pandemic setting, rapid formation of protective immunity is key. It is therefore important that both Pandemrix and αMHCII-HA were able to induce moderate protection against a lethal viral challenge already 8 days post a single vaccination. Interestingly, the DNA vaccine demonstrated a slight increase in survival as compared to Pandemrix, but significantly reduced morbidity. As expected, TIV did not induce any sign of immune resistance to challenge only 8 days after vaccination. At this time point, neither vaccine conferred sterilizing immunity against influenza. However, strong cytotoxic T-cell responses elicited by the MHCII targeted DNA vaccine are likely the underlying reason for the observed reduced morbidity in this vaccine group, as demonstrated by the significantly lower weight loss (**Figure 2**). This explanation was supported by observations of increased levels of CD4 and CD8 effector memory T cells after DNA vaccination. The clear differences in cytotoxicity observed *ex vivo* was also supported by the T cell cytokine profiles, with key cytokines such as IFN-γ, IL-2, and TNF-α elevated after DNA immunization both for the CD8 and CD4 T-cell compartment. The T cell responses raised at day 8 post vaccination were, however, not sufficient for protection against a different strain of influenza H1N1 (**Figures 2G, H**). However, we have previously found that the cellular immune responses induced by αMHCII-HA can protect against antigenically variable strains at 4 weeks after a single DNA vaccination (22, 50).

The hemagglutination inhibition (HI) assay is currently at the core for regulatory assessments of influenza vaccine immunogenicity (51). It evaluates the ability of antibodies to prevent virus from binding to red blood cells. Concerns about considering the HI titer a lone predictor of vaccine efficacy have been raised (52), especially for strains with a pandemic potential (53). During the past 20 years, several influenza subtypes (e.g. H5, H7, H9, and H10) have been demonstrated to breach the zoonotic barrier (54). The influenza virus is prone to antigenic drift, potentially hampering the efficacy of vaccine induced strain specific and neutralizing antibodies. Vaccines against influenza pandemics should therefore ideally be able to raise a combination of protective antibodies and T cell responses, offering at least some protection also against strain variants that may emerge. The ability of the MHCII-targeted DNA vaccine to raise a broader type of immune response, including both strong antibody responses and T cells, is encouraging in this respect.

For pandemic preparedness, one should consider the contribution from cytotoxic T cells induced solely by vaccines or in combination with pre-existing immunity. T cells often react to conserved epitopes that are shared among many different strains or even subtypes of influenza, offering immune resistance in the absence of effective antibodies (55). An ideal vaccine for pandemic preparedness should therefor activate both arms of the immune system and induce neutralizing antibodies as well as cytotoxic T cell responses. T cell mediated immunity cannot confer sterilizing immunity, but the broader responses to more conserved epitopes in the virion may prevent progression to severe morbidity or mortality. Thus, it may be important to establish T cell based correlates of protection against disease for improved evaluation and approval of influenza vaccines.

In summary, DNA vaccines targeting HA to MHCII molecules demonstrated comparable antibody responses and efficacy to Pandemrix in a mouse model. A noteworthy difference between these two vaccines was the cytotoxic T-cell response after vaccination with αMHCII-HA, that likely improved symptomatic disease at an early time-point after a single vaccination. Due to the many advantages of the DNA vaccine format over egg-based split virus vaccines, these data confirms the relevance of DNA vaccines as an attractive approach for pandemic preparedness.

## Data Availability Statement

The original contributions presented in the study are included in the article/supplementary material. Further inquiries can be directed to the corresponding author.

## Ethics Statement

The animal study was reviewed and approved by Norwegian Food Safety Authority.

## Author Contributions

GG, SM, TKA, and BB conceived and designed experiments. TKA, JB, SM, FO, and GG performed and analyzed experiments. TKA and GG wrote the paper. All authors edited and commented on the paper. All authors contributed to the article and approved the submitted version.

## Funding

The work has been funded by the Research Council of Norway, as well as Helse Sør-Øst.

## Conflict of Interest

GG and BB are inventors on a patent applications filed on an HLAII-specific targeting moiety according to institutional rules through the TTO offices of the University of Oslo and Oslo University Hospital. Further, BB is inventor of the core patent of Vaccibody AS, and hold shares in the company.

The remaining authors declare that the research was conducted in the absence of any commercial or financial relationships that could be construed as a potential conflict of interest.

## Publisher’s Note

All claims expressed in this article are solely those of the authors and do not necessarily represent those of their affiliated organizations, or those of the publisher, the editors and the reviewers. Any product that may be evaluated in this article, or claim that may be made by its manufacturer, is not guaranteed or endorsed by the publisher.
